# Inhibition of renal cell carcinoma angiogenesis and growth by antisense oligonucleotides targeting vascular endothelial growth factor

**DOI:** 10.1038/sj.bjc.6600416

**Published:** 2002-07-15

**Authors:** W Shi, D W Siemann

**Affiliations:** Department of Pharmacology and Experimental Therapeutics, University of Florida, Box 100267, 1600 SW Archer Road, Gainesville, FL 32610, USA; Department of Radiation Oncology, Shands Cancer Centre, University of Florida, Box 100385, 2000 SW Archer Road, Gainesville, FL 32610, USA

**Keywords:** renal cell carcinoma, vascular endothelial growth factor, angiogenesis, antisense oligodeoxynucleotides

## Abstract

Angiogenesis is critical for growth and metastatic spread of solid tumours. It is tightly controlled by specific regulatory factors. Vascular endothelial growth factor has been implicated as the key factor in tumour angiogenesis. In the present studies we evaluated the effects of blocking vascular endothelial growth factor production by antisense phosphorothioate oligodeoxynucleotides on the growth and angiogenic activity of a pre-clinical model of renal cell carcinoma (Caki-1). *In vitro* studies showed that treating Caki-1 cells with antisense phosphorothioate oligodeoxynucleotides directed against vascular endothelial growth factor mRNA led to a reduction in expressed vascular endothelial growth factor levels sufficient to impair the proliferation and migration of co-cultured endothelial cells. The observed effects were antisense sequence specific, dose dependent, and could be achieved at a low, non-toxic concentration of phosphorothioate oligodeoxynucleotides. When vascular endothelial growth factor antisense treated Caki-1 cells were injected into nude mice and evaluated for their angiogenic potential, the number of vessels initiated were approximately half that induced by untreated Caki-1 cells. To test the anti-tumour efficacy of vascular endothelial growth factor antisense, phosphorothioate oligodeoxynucleotides were administrated to nude mice bearing macroscopic Caki-1 xenografts. The results showed that the systemic administration of two doses of vascular endothelial growth factor antisense phosphorothioate oligodeoxynucleotides given 1 and 4 days after the tumours reached a size of ∼200 mm^3^ significantly increased the time for tumours to grow to 1000 mm^3^.

*British Journal of Cancer* (2002) **87**, 119–126. doi:10.1038/sj.bjc.6600416 www.bjcancer.com

© 2002 Cancer Research UK

## 

Angiogenesis, a complex multi-step process involving the formation of new blood vessels from pre-existing ones, is tightly regulated by both positive and negative regulatory factors (Risau, 1997). These regulators, which included pro-angiogenic factors such as basic fibroblast growth factor (bFGF) (Wang and Becker, 1997), angiogenin (Gho and Chae, 1997) and vascular endothelial growth factor (VEGF) (Kim *et al*, 1993; Asano *et al*, 1995; Borgstrom *et al*, 1996; Cheng *et al*, 1996; Saleh *et al*, 1996), as well as angiostatic peptides such as endostatin (O'Reilly *et al*, 1997; Perletti *et al*, 2000), angiostatin (O'Reilly *et al*, 1994a,b) and thrombospondin (Folkman and Shing, 1992; Folkman, 1995) are potential targets for anti-angiogenic therapy of solid tumours (Folkman, 1971; Bicknell and Harris, 1992; Harris *et al*, 1994; Denekamp, 1999).

Vascular endothelial growth factor is an endothelial cell specific mitogen, secreted as a 45 kDa homo dimer protein. There are five human isoforms derived from alternative splicing (VEGF 121, 145, 165, 189, 206) (Houck *et al*, 1991). VEGF121 and VEGF165 are the only soluble isoforms and also the most abundant, with VEGF165 being the most powerful stimulator of endothelial cell proliferation (Soker *et al*, 1997). VEGF165 is commonly expressed in a wide variety of human and animal tumours (Hanahan and Folkman, 1996) and has been shown to induce angiogenesis both *in vitro* and *in vivo* (Leung *et al*, 1989; Plate *et al*, 1992). It is currently believed that this diffusible molecule is probably a key mediator of tumour angiogenesis (Ferrara, 1999). Indeed, the expression of VEGF has been related to fundamental features of tumours, such as growth rate (Kim *et al*, 1993), microvessel density (Toi *et al*, 1994) and vascular architecture (Drake, 1999) as well as the development of tumour metastasis (Weidner *et al*, 1991). A correlation between VEGF expression and survival has been noted in some cancer patients (Maeda *et al*, 1996; Gasparini *et al*, 1997).

In light of its role in tumour angiogenesis, VEGF may be an attractive target for anti-angiogenic therapeutic interventions applied to the treatment of cancer. Renal cell carcinoma (RCC) may be an excellent site to investigate VEGF targeted anti-angiogenic therapies. RCC is the most common malignancy of the kidney in adults and accounts for about 2% of all adult malignancies (McLaughlin and Lipworth, 2000). Histopathologic evaluations of RCC reveal it to be a highly vascularised neoplasm demonstrating clear evidence of abundant angiogenesis and abnormal blood vessel development (Yoshino *et al*, 2000). Not surprisingly, several studies have pointed to an important role for pro-angiogenic growth factors in RCC. VEGF has been shown to be expressed in renal cell carcinoma tissues and renal cell carcinoma cell lines (Sato *et al*, 1994; Wang and Becker, 1997; Paradis *et al*, 2000). Serum levels of VEGF often are elevated in RCC patients (Sato *et al*, 1999) and VEGF mRNA levels in renal cell carcinoma have been reported to be higher than those found in surrounding normal tissues (Takahashi *et al*, 1994; Berger *et al*, 1995). In addition, elevated serum/urine VEGF levels have been shown to associated with malignant progression and poor treatment outcome (Sato *et al*, 1994; Baccala *et al*, 1998; Tomisawa *et al*, 1999; Ishizuka *et al*, 2000; Jacobsen *et al*, 2000). Taken together, these findings suggest that VEGF is one of the important factors involved in the angiogenesis of RCC.

In the present study we evaluated the anti-angiogenic and anti-tumour effects of VEGF antisense phosphorothioate oligodeoxynucleotides (PS-ODNs) in a pre-clinical model of human RCC (Caki-1).

## MATERIALS AND METHODS

### Cell culture

The clear cell RCC cell line Caki-1 was a gift from Dr Susan Knox (Stanford University). Caki-1 cells were grown in Dulbecco's modified minimum essential medium (DMEM Invitrogen, Grand Island, NY, USA) supplemented with 10% foetal bovine serum (FBS, Invitrogen, Grand Island, NY, USA), 1% penicillin-streptomycin (Invitrogen, Grand Island, NY, USA) and 1% 200 mmol l^−1^ L-glutamine (Invitrogen, Grand Island, NY, USA). The mouse heart endothelial cell line (MHE) was a gift from Dr Robert Auerbach (University of Wisconsin). MHE cells were grown in DMEM supplemented with 10% heat inactivated foetal bovine serum, 1% penicillin-streptomycin and 1% 200 mmol l^−1^ L-glutamine. Human microvascular endothelial cell from the lung (HMVEC-L) cells were obtained from Clonetics (San Diego, CA, USA). HMVEC-L cells were grown in EBM-2-MV (Clonetics, San Diego, CA, USA) supplemented with 5% FBS.

### Caki-1 xenografts

Female nude mice (NCR, nu/nu), age 6–8 weeks were maintained under specific-pathogen-free conditions (University of Florida Health Science Centre) with food and water supplied *ad libitum*. Animals were inoculated subcutaneously in a single flank with 5×10^6^ tumour cells. When the tumours reached a size ∼200 mm^3^, animals were randomly assigned to the different treatment groups. All animal experiments have been carried out with ethical committee approval. The ethical guidelines that were followed meet the standards required by the Cancer Research UK guidelines (Workman *et al*, 1998).

### Phosphorothioate oligodeoxynucleotides (PS-ODNs)

Antisense and control PS-ODNs (20-mers) were custom synthesised by Geno Mechanix (Alachua, FL, USA). PS-ODNs V515 was complementary to 5′ UTR just up-stream of the translation start site (AUG codon) of VEGF mRNA: 5′-CTC ACC CGT CCA TGA GCC CG-3′. A scramble sequence: 5′-CAC CCT GCT CAC CGC ATG GC-3′; sense sequence: 5′-CGG GCT CAT GGA CGG GTG AG-3′ and an inverted sequence: 5′-GCC CGA GTA CCT GCC CAC TC-3′, were used as PS-ODNs controls. All PS-ODNs were suspended in sterile and endotoxin free water at a concentration of 1 mM, aliquoted and stored at −20°C.

### DOTAP : DOPE liposome preparation

Cationic liposomes were prepared using the method described by Tang and Hughes (1999). Briefly, cationic lipid 1,2-dioleoyloxy-3-(trimethylammonium) propane (DOTAP) was dissolved in chloroform and mixed with a helper lipid 1,2-dioleoyl-3-sn-phosphatidylethanolamine (DOPE) (Avanti Polar-Lipids, Alabaster, AL, USA) at a molar ratio of 1 : 1. The mixture was evaporated to dryness in a round-bottomed flask using a rotary evaporator at room temperature. The resulting lipid film was dried by nitrogen for an additional 10 min to evaporate any residual chloroform. The lipid film was re-suspended in sterile water to a final concentration of 1 mg ml^−1^ based on the weight of cationic lipid. The resultant mixtures were shaken in a water bath at 35°C for 30 min. The suspensions were then sonicated using a Sonic Dismembrator (Fisher Scientific, Pittsburgh, PA, USA) for 1 min at room temperature to form homogenised liposomes. The particle-size distribution of liposomes was measured using a NICOMP 380 ZLS instrument (Santa Barbara, CA, USA). The average particle diameter was 144.0±77.0 nm. Liposomes were stored at 4°C and used within 3 months.

### VEGF enzyme immunoassay

Caki-1 cells (1×10^5^) were set in 60 mm dishes and allowed to attach overnight. The medium then was removed and replaced with PS-ODNs in serum free medium with liposome (DOTAP : DOPE) and incubated for 5 h. Fresh medium containing 10% FBS then was added. After 24 h of incubation the VEGF concentration was determined in the medium using a human VEGF immunoassay kit (R & D Systems, Minneapolis, MN, USA).

### VEGF relative quantitative RT–PCR

Caki-1 cells were set at 3×10^5^ in 100 mm dishes and allowed to attach overnight. The cells were then treated with 1 μM VEGF antisense or control PS-ODNs as described. Twenty-four hours later the cells were collected and the total RNA was isolated using a RNeasy Mini Kit (Qiagen, Valencia, CA, USA) and RNA concentrations were determined by UV spectrophotometry. A 2 μg total RNA sample was used to reverse synthesize cDNA using Superscript II reverse transcriptase (Invitrogen, Grand Island, NY, USA). A 2.5 μl aliquot of the reverse transcriptase reaction product then was used for the PCR reaction. VEGF PCR reactions were carried out with a VEGF gene specific relative RT–PCR Kit (Ambion, Austin, TX, USA). The PCR reactions were run 22 cycles (denature 94°C 30 s, anneal 60°C 60 s, extension 72°C 60 s) in a DNA Engine 200 (MJ research, Waltham, MA, USA). PCR products then were run in 2% agrose gel and stained by ethidium bromide. The gels were visualised and analysed (Gel Doc 2000 gel documentation system, Bio-Rad, Hercules, CA, USA).

### Co-culture assay

Transwell (Corning, Corning, NY, USA) 6-well dishes with a membrane pore size of 0.4 μM were used. Caki-1 cells were seeded at 5×10^4^ in the transwell inserts and MHE or HMVEC-L cells were plated at 5×10^4^ per well in the 6-well dishes and allowed to attach overnight. The Caki-1 cell medium was then replaced with serum free medium containing 1 μM V515 PS-ODNs or control PS-ODNs liposome complexes (DOTAP : DOPE). After 5 h of treatment, medium containing 10% heat inactivated FBS was added to yield a final FBS concentration of 2.5%. The transwells containing treated Caki-1 cells were assembled with 6-well dished containing MHE and HMVEC-L cells and incubated at 37°C for 48 h at which time the numbers of MHE or HMVEC-L cells were determined by haemocytometer count.

### Migration assay

Caki-1 cells were set at 1×10^5^ per well in 24-well dishes and allowed to attach overnight. The Caki-1 cells then were treated with 1 μM control or V515 PS-ODNs for 24 h. HTS FluoroBlok inserts (Becton Dickinson, Franklin Lakes, NJ, USA) with a pore size of 8.0 μm were assembled into the 24-well dish with the Caki-1 cells. MHE or HMVEC-L cells were grown in T-150 flasks to about 80% confluence. The endothelial cells were stained in medium containing 10 μg ml^−1^ Di-I (Molecular Probes, Eugene, OR, USA) for 24 h, washed four times with PBS, collected, added into the FluoroBlok inserts (5×10^4^ MHE or HMVEC-L) and incubated for 24 h. The number of migrated endothelial cells then were determined by direct measurement of the fluorescence in the bottom well using a CytoFluor 4000 plate reader (Perceptive BioSystems, St. Paul, MN, USA).

### Intradermal angiogenesis assay

Caki-1 cells (5×10^4^) were inoculated intradermally in a volume of 10 μl at four sites on the ventral surface of nude mice. One drop of 0.4% trypan blue was added to the cell suspension, which making it lightly coloured, simplifying subsequent location of the sites of injection. Three days later the mice were killed, the skin carefully separated from the underlying muscle and the number of vessels counted using a dissecting microscope (Sidky and Auerbach, 1976). Scoring of all of the reaction areas was carried out at the same magnification (×5) and only vessels readily detected at this magnification were counted. The sites of injection, recognised by local swelling and blue staining, were exposed by carefully removing fat or other tissue covering the area. All vessels that touched the edge of the tumour inoculates were counted. All the animals in the experiments were pre-coded and vessel counts in each animal were scored twice. The resultant data points for each treatment group were pooled for statistical analysis (Wilcoxon rank sum test).

### PS-ODNs up-take in tumour

FITC labelled control PS-ODNs were mixed with DOTAP : DOPE in 200 μl 5% dextrose and injected into Caki-1 xenograft bearing mice via the tail vein at a dose of 20 mg kg^−1^. Three hours later, the mice were killed by CO_2_ asphyxiation, the tumours removed, frozen in liquid nitrogen and 20 μm sections were cut. The sections were photographed using a Zeiss Axioplan 2 Florescence Microscope (Zeiss, Thornwood, NY, USA) within 3 days.

### VEGF Western blot

VEGF antisense PS-ODNs V515 was injected tail vein at a dose of 10 mg kg^−1^. At various times after injection (24, 48 and 72 h), the mice were killed, the tumours excised and frozen in liquid nitrogen. The tumours were then homogenised (Dounce tissue grinder, Wheaton, Millville, NJ, USA) and the homogenates were lysed on ice for 30 min with 1 ml of hypotonic buffer (20 mM Tris-HCl, pH 6.8, 1 mM MgCl_2_, 2 mM EGTA, 0.5% Nonidet P-40, 2 mM Phenylmethanesulphonyl fluoride (PMSF), 200 u ml^−1^ Approtinin, 2 μg ml^−1^ leupetin) (Giannakakou *et al*, 1998) per 0.1 g tissue. Following a brief but vigorous vortex the samples were centrifuged at 14 000 r.p.m. for 10 min at 4°C. A 30 μl aloquot of each sample was mixed with 10 μl 4× SDS–PAGE sample buffer (0.3 M Tris-HCl, pH 6.8, 45% glycerol, 20% β-mercaptoethanol, 9.2% SDS and 0.04 g per 100 ml bromophenol blue) and heated at 100°C for 10 min. Thirty μl of each sample was then analysed by SDS–PAGE on a 12% separating gel and 3% stacking gel. Following transfer, the membrane was immunoblotted using a VEGF primary antibody (Sigma, Saint Louis, MS, USA) 1 : 1000 diluted in antibody solution (3% dry milk, 25 mM Tris, pH 7.5, 0.5 M NaCl, 0.05% Tween 20) overnight at 4°C. After washing, a secondary antibody labelled with horseradish peroxidase was applied and incubated at room temperature for 1 h. Protein bands were visualised and densitometry was performed.

### Tumour growth delay assay

Once the Caki-1 xenografts reached a size of ∼200 mm^3^, animals were assigned randomly to various treatment groups. V515 or control PS-ODNs were administrated via the tail vein with DOTAP : DOPE liposomes at a dose of 5 mg kg^−1^ or 10 mg kg^−1^ on day 1 and day 4. Tumours were measured using callipers and volumes were approximated by the formula, volume=*1/6*π*ab^2^*, with *a* and *b* represent two perpendicular tumour diameters. The times for the tumours in the various treatment groups to grow from 200 mm^3^ to 1000 mm^3^ were recorded and compared (Wilcoxon rank sum test).

## RESULTS

**Figure 1:**
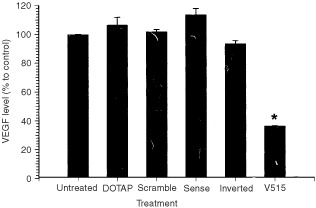
VEGF levels in culture medium of Caki-1 tumour cells treated with different antisense PS-ODNs. The cells were either untreated (Untreated) or treated with delivery vehicle only (DOTAP), a 1 μM dose of control PS-ODNs sequences (Scramble, Sense and Inverted) or a 1 μM dose of antisense PS-ODNs targeted to a specific sequence of VEGF mRNA (V515). Each bar represents the mean±s.e. of three independent experiments. The star indicates a significant difference from the untreated or control treated groups (*P*<0.05, student's *t*-test). The 100% VEGF expression level of the untreated group corresponds to ∼850 pg ml^−1^ 10^6^ cells^−1^.

**Figure 2:**
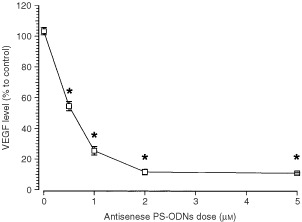
VEGF levels in the culture medium of Caki-1 tumour cells treated with different doses (0.5, 1, 2, 5 μM) of VEGF antisense PS-ODNs (V515). The 0 dose group was treated with scramble control PS-ODNs (5 μM). Each square represents the mean±s.e. of three independent experiments. The stars indicate significant differences from 0 dose (*P*<0.05, student's *t*-test).

**Figure 3:**
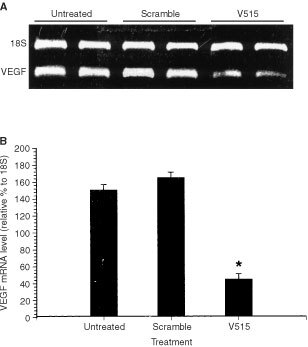
Message RNA levels in Caki-1 cells which were either untreated or treated with a 1 μM dose of scramble control sequence or VEGF antisense PD-ODNs (V515). (**A**) Representative relative RT–PCR results, each group was performed in duplicate; 18s indicates the RT–PCR amplification band of 18s ribosomal RNA. (**B**) Relative VEGF mRNA levels of Caki-1 cells treated with different PS-ODNs. The values were obtained by dividing the densitometer reading of the VEGF band by that of the 18s band. Each bar is the mean±s.e. of three experiments. The star indicates a significant difference (*P*<0.05, student's *t*-test).

The ability to down-regulate VEGF expression by antisense PS-ODNs treatment in Caki-1 tumour cells was first evaluated *in vitro*. The results showed that after 24 h treatment with 1 μM VEGF antisense PS-ODNs (V515) delivered by cationic liposome (DOTAP : DOPE), the medium VEGF level was significantly reduced to ∼35% that found in the control untreated group (from a normal of 850 pg ml^−1^ 10^6^ cells^−1^ to 300 pg ml^−1^ 10^6^ cells^−1^) (Figure 1

). This effect was sequence, and target region specific. Treating Caki-1 cells with liposome vehicles (DOTAP: DOPE) or control scramble PS-ODNs did not affect VEGF levels. Similarly, treatment with sense or inverted sequence PS-ODNs failed to reduce VEGF expression. PS-ODNs treatment did not affect Caki-1 cell viability and proliferation. This repression of VEGF expression by V515 was dose dependent (Figure 2

). For example, a 24 h treatment with 0.5 μM, reduced the medium VEGF level to 56% of control (*P*<0.01) whereas a 1 μM dose down-regulated the VEGF level to 22% of control (*P*<0.05). VEGF mRNA levels in different PS-ODNs treatment groups also were determined (Figure 3

). The results indicated a marked inhibition of VEGF mRNA after treatment with V515 which was absent in cells treated with scramble PS-ODNs.

**Figure 4:**
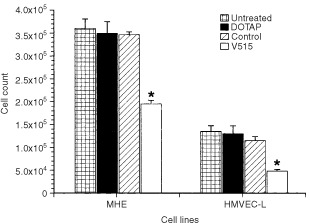
Effect of co-culturing Caki-1 tumour cells on the growth of endothelial cells (MHE and HMVEC-L). Caki-1 cells were untreated or pre-treated with DOTAP liposome vehicle, 1 μM scramble control PS-ODNs or 1 μM V515 antisense PS-ODNs. Each bar represents the mean±s.e. of three independent experiments. Stars indicate significant difference (*P*<0.05, student's *t*-test) from the untreated group.

Since the goal of VEGF antisense therapy is to inhibit cancer cell induced angiogenic signalling, experiments then were designed to evaluate the impact of reducing tumour cell expression of VFGF by antisense PS-ODNs treatment on endothelial cell growth and migration. Because the ultimate goal was to examine the efficacy of VEGF antisense treatment in a human tumour model grown in a mouse host, we evaluated the effect on both human (HMVEC-L) and mouse (MHE) endothelial cells. Transwell co-culture systems were used to mimic the *in vivo* paracrine interaction between tumour cells and endothelial cells. Caki-1 tumour cells were grown in transwells with 0.4 μm membrane pores. These were chosen to allow the exchange of growth factors but without direct cell–cell interactions. The effects of pretreating Caki-1 tumour cells with VEGF antisense PS-ODNs on endothelial cell proliferation then were determined (Figure 4

). The results showed that compared to untreated Caki-1 cells, Caki-1 cells pre-treated with V515 significantly inhibited both HMVEC-L and MHE cell proliferation. Once again, treating Caki-1 cells with a variety of control PS-ODNs had no effect on HMVEC-L or MHE cell growth.

**Figure 5:**
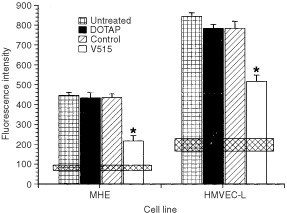
Impact of co-culturing Caki-1 tumour cells on ability of MHE and HMVEC-L cells to migrate. Caki-1 cells were untreated or pre-treated with DOTAP liposome vehicle, a 1 μM dose of scramble control or a 1 μM dose of V515 antisense PS-ODNs. Each bar represents the mean±s.e. of three experiments. The stars indicate a significant difference (*P*<0.05, student's *t*-test) from the untreated groups. The horizontal bars show the levels of spontaneous migration of endothelial cells cultured alone; the bar widths correspond to the mean values±1 s.e.

To test whether a reduction in VEGF expression by tumour cells could affect endothelial cell migration, HMVEC-L or MHE cells were stained with 10 μg ml^−1^ Di-I for 24 h and added into Fluoroblok inserts placed into 24 well dishes containing Caki-1 tumour cells treated with either control or V515. The number of pre-labelled endothelial cells which migrated through the 8 μm pore size membranes in a 24 h period were quantified by determining the fluorescence intensity in the bottom well. The results showed (Figure 5

) that 24 h after co-culturing the two cell populations ∼35% (*P*<0.05) and 27% (*P*<0.05) fewer MHE or HMVEC-L cells respectively migrated through the membrane in the presence of V515 treated Caki-1 cells compared to untreated or scramble control PS-ODNs treated Caki-1 cells.

**Figure 6:**
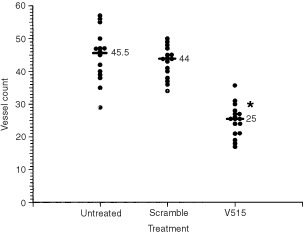
Number of blood vessels induced 3 days after injecting 5×10^4^ Caki-1 cells intradermally in nude mice. Caki-1 cells were either untreated or pre-treated with a 1 μM dose of PS-ODNs for 3 h. The Scramble group refers to cells pre-treated with scramble control PS-ODNs, whereas the V515 group represents Caki-1 cells pre-treated with VEGF antisense PS-ODNs (V515). Each datum point represents one injection site, the bars show the median of 16 sites in each group. The V515 treated group was significantly different from the untreated or scramble control groups (*P*<0.05, Wilcoxon rank sum test).

Although, the *in vitro* studies indicated that treating Caki-1 tumour cells with VEGF mRNA targeted antisense PS-ODNs down-regulated VEGF protein production sufficiently to affect the proliferation and migration of endothelial cells, it was important to demonstrate that such treatments also could affect Caki-1 cell induction of angiogenesis *in vivo*. To examine this possibility Caki-1 cells that had been treated with V515 or control PS-ODNs were injected intradermally and the number of vessels induced were counted 3 days later (Figure 6

). While untreated Caki-1 cells and control PS-ODNs treated Caki-1 cells had very similar angiogenic potency *in vivo* (both groups induced ∼44–46 new vessels in the assay period), the angiogenic potential of Caki-1 cells that had been pre-treated with V515 antisense PS-ODNs (V515) was found to be significantly impaired; only ∼25 new blood vessels were observed.

**Figure 7:**
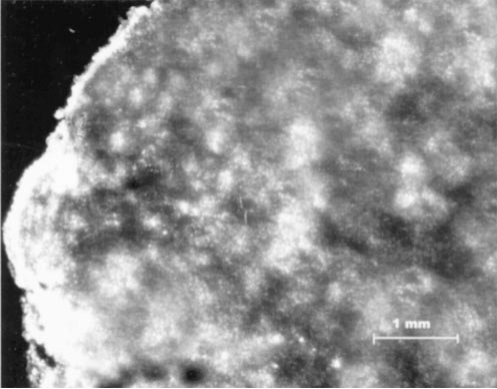
Uptake and distribution of FITC-labelled PS-ODNs in Caki-1 xenografts determined 3 h after an i.v. injection of a 20 mg kg^−1^ dose. PS-ODNs were prepared with DOTAP : DOPE. Sections were 20 μm.

**Figure 8:**
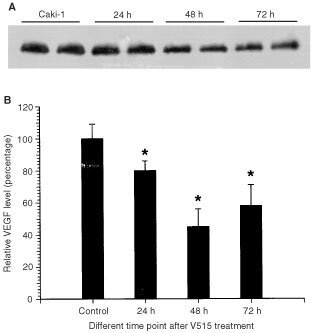
VEGF protein levels in Caki-1 tumours at different time points after treatment of 10 mg kg^−1^ VEGF antisense PD-ODNs (V515). (**A**) Representative VEGF Western blot results, showing two tumour samples of each group; (**B**) Relative VEGF protein levels of Caki-1 tumours treated VEGF antisense PD-ODNs (V515). Each bar is the mean±s.e. of six tumours. The star indicates a significant difference (*P*<0.05, student's *t*-test).

To evaluate the tumour up-take of PS-ODNs delivered by cationic liposome (DOTAP : DOPE). FITC labelled PS-ODNs were mixed with cationic liposome DOTAP : DOPE in 5% dextrose and were injected *via* the tail vein at a dose of 20 mg kg^−1^ into Caki-1 xenograft-bearing nude mice. Frozen sections prepared 3 h later showed the FITC labelled PS-ODNs to be efficiently delivered to the tumour (Figure 7

). The heterogeneous PS-ODNs uptake likely reflects the inhomogeneous distribution of blood vessels in the tumour. To further confirm the antisense effect of V515 *in vivo*, tumour samples were collected at various times after V515 injection. Western blot analysis of these samples showed significant reductions in VEGF levels at 24, 48 and 72 h, with the maximum depression occurring at 48 h after treatment (Figure 8

). These findings clearly indicate the delivery of VEGF antisense to the tumour would result in a reduction in VEGF expression levels.

**Figure 9:**
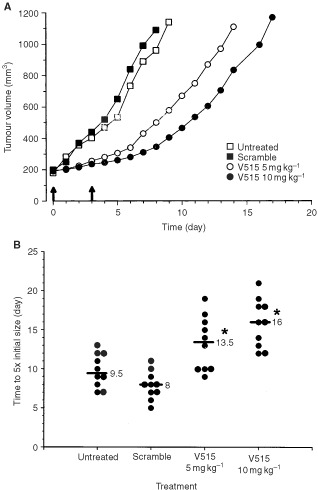
(**A**) Growth of median RCC Caki-1 tumours in nude mice treated systemically with antisense PS-ODNs against VEGF. Mice were untreated, treated with 10 mg kg^−1^ scramble PS-ODNs, 5 mg kg^−1^ VEGF antisense V515, or 10 mg kg^−1^ VEGF antisense V515 at the time indicated by arrows. Each group consisted of 10 animals. (**B**) Tumour response of Caki-1 xenografts treated systemically with antisense PS-ODNs targeted to VEGF mRNA. Scramble control (Scramble) or VEGF antisense (V515) PS-ODNs were administrated with cationic liposome (DOTAP : DOPE) via the tail vein 1 and 4 days after the tumours reached a size of ∼200 mm^3^. Liposome administration alone had no effect on Caki-1 tumour growth (data not shown). Each circle represents a single tumour; the bars show the response of the median tumour in each group of 10 mice. The stars indicate significant differences (*P*<0.05, Wilcoxon rank sum test) from the untreated and scramble control groups.

Subsequent experiments were undertaken to determine the antitumour efficacy of V515 antisense PS-ODNs when delivered systemically by examining the effect of such treatments on Caki-1 tumour growth. Caki-1 xenografts-bearing mice were treated with two doses of VEGF antisense PS-ODNs V515 (5 or 10 mg kg^−1^) 1 and 4 days after the tumours reached a size of ∼200 mm^3^. The time for the tumours to grow from 200 mm^3^ to 1000 mm^3^ then was recorded (Figure 9

). The data showed that the median time for the tumours to grow to five times the starting size was significantly prolonged in the V515 antisense PS-ODNs (V515) treated groups. Administrating two doses of 5 mg kg^−1^ caused a growth delay of ∼5.5 days (*P*<0.05, Wilcoxon rank sum test) while treatment with two 10 mg kg^−1^ doses led to a tumour growth delay of ∼8 days. The latter treatment therefore resulted in an approximately doubled the response of the tumours compared to the tumours of untreated or scramble PS-ODNs treated mice. No toxicity of such antisense PS-ODNs treatment was observed. This included no significant weight loss, no abnormal movements or behaviour and no loose stools.

## DISCUSSION

Anti-angiogenesis treatment strategies represent a new approach to cancer management. Given that solid tumours cannot progress effectively without the generation of new blood vessels, various tacks have been taken to interfere with tumour angiogenesis. One possible target which has received considerable attention is the pro-angiogenic factor VEGF. VEGF can induce endothelial cell proliferation and migration *in vitro* (Hanahan and Folkman, 1996; Soker *et al*, 1997) and angiogenesis *in vivo* (Leung *et al*, 1989; Plate *et al*, 1992). Its expression level has been associated with a variety of tumours and correlated to treatment outcome (Maeda *et al*, 1996; Gasparini *et al*, 1997). To date, attempts to abrogate the angiogenic activity of VEGF have focused on inactivating VEGF through the use of antibodies (Kim *et al*, 1993; Mordenti *et al*, 1999) and soluble receptors (Lin *et al*, 1998), inhibiting VEGF receptor tyrosine kinases (Hennequin *et al*, 1999) or suppressing VEGF message (Ellis *et al*, 1996; Smyth *et al*, 1997; Nguyen *et al*, 1998). The latter relied on antisense oligonucleotides or antisense RNA (Eguchi *et al*, 1991; Mercola and Cohen, 1995; Phillips and Zhang, 2000) to modulate gene expression by disrupting RNA expression. While the inhibition of VEGF expression by vector mediated gene transfer of antisense RNA has been shown to lead to growth delays in several tumour models (Folkman and Shing, 1992; Belletti *et al*, 1999; Kang *et al*, 2000; Nakashima *et al*, 2000), the only reports of *in vivo* efficacy when using VEGF antisense oligonucleotides occurred in VEGF dependent tumour models (Masood *et al*, 1997, 2001). In the present studies, we used a VEGF independent tumour model of RCC (VEGF-R negative) to evaluate the *in vitro* and *in vivo* efficacy of a different VEGF antisense PS-ODNs sequence (V515). Antisense oligodeoxynucleotide technology provides an approach for inhibiting gene expression with target specificity as a particular advantage (Stein and Cheng, 1993; Engelhard, 1998). Antisense oligonucleotides are easy to produce in large quantities which make them potentially more practical than antisense RNA vector delivery approaches.

In the present study, we investigated the anti-angiogenic and anti-tumour effects of VEGF antisense PS-ODNs in a VEGF independent tumour model of RCC. *In vitro* experiments showed that the inhibition of VEGF production in Caki-1 tumour cells by antisense PS-ODNs treatment significantly reduced the ability of co-cultured endothelial cells to proliferate (Figure 4) and migrate (Figure 5). To minimise interference of other growth factors in the serum, these studies were carried out under reduced serum conditions. Importantly, suppressing tumour cell expression of VEGF message impaired angiogenic responses in both human and mouse endothelial cells. Subsequent *in vivo* studies demonstrated that treating Caki-1 tumour cells with VEGF antisense PS-ODNs significantly impaired their ability to elicit an angiogenic response when injected intradermally (Figure 6). These results not only support the role of VEGF as an important pro-angiogenic growth factor in Caki-1 cell induced angiogenesis, but also clearly suggest that inhibition of cancer cell VEGF expression may ultimately impact tumour growth. This belief was born out in experiments evaluating the *in vivo* anti-tumour efficacy of systemic administration of VEGF antisense PS-ODNs which showed that substantial tumour growth delays could be achieved with such treatment (Figure 9).

The results of this study indicate a key role for the VEGF signalling pathway in renal cell carcinoma angiogenesis. Treatment with VEGF antisense PS-ODNs to down regulate VEGF production was found to be effective at impairing the Caki-1 angiogenic signalling both *in vitro* and *in vivo*. Most importantly the systemic administration of VEGF antisense PS-ODNs to mice bearing macroscopic tumours resulted in significant inhibition of the growth of VEGF independent RCC tumour model. Taken together, these findings suggest that antisense PS-ODNs targeted to VEGF may have utility in the management of renal cell carcinoma either alone or in conjunction with conventional anti-cancer therapies.
